# Study on the Drying Technology of Tomato Pulp with Phytoene, Phytofluene and Lycopene Retention as Inspection Indexes

**DOI:** 10.3390/foods11213333

**Published:** 2022-10-24

**Authors:** Liying Li, Cheng Yang, Jian Zhang, Lianfu Zhang

**Affiliations:** 1 State Key Laboratory of Food Science and Technology, Jiangnan University, 1800 Lihu Avenue, Wuxi 214122, China; 2 College of Food, Shihezi University, Beisi Road, Shihezi 832003, China

**Keywords:** phytoene (PT), phytofluene (PTF), lycopene (LYC), drying technology, retention rate

## Abstract

The objective was to design a feasible drying method to increase the retention rates of phytoene (PT), phytofluene (PTF) and lycopene (LYC) in tomato powder. The method was to compare the effects of vacuum freeze-drying (FD), vacuum drying (VD) and hot-air drying (HAD) technologies on tomato pulp rich in PT, PTF and LYC. When dried by HAD, the retention rates of PT, PTF and LYC decreased significantly (*p* < 0.05) only when the water content decreased from 30% to 3.5%. When dried by VD, the temperatures had no significant effect on the retention rates, and only alkaline conditions (pH = 9), Fe^3+^ and Al^3+^ could significantly reduce the retention rates (*p* < 0.05). Therefore, a combined drying process (CDP) was designed: before the water content decreased to 50%, HD (60 °C) technology was used; then, the paste was dried via VD (80 °C, 0.08 MPa) technology till the water content reached 5 ± 2%; loading weight was 40 g (thinkness 5.70 mm) for each batch. Compared with VD alone, the CDP technology improved the retention rates of PT and LYC by 12% and 36%, respectively, while PTF decreased by only 6%.

## 1. Introduction

**Figure 1:**
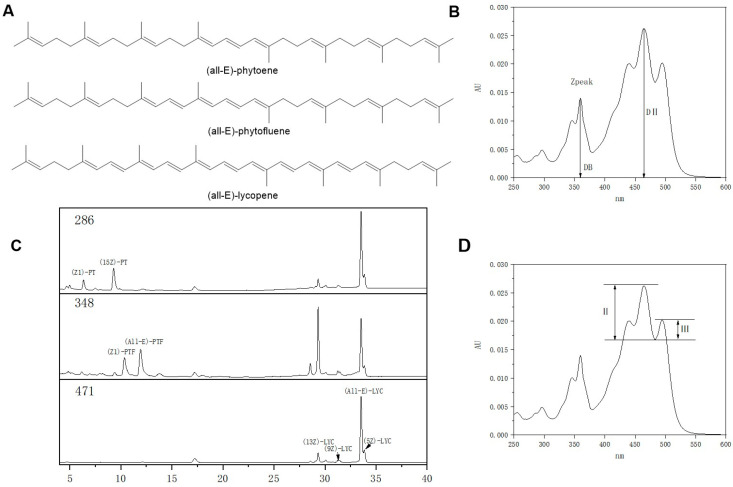
Structures of phytoene (PT), phytofluene (PTF) and lycopene (LYC), and isomer determination methods. (**A**) Structures of PT, PTF and LYC. (**B**) The calculation method of Q value (DB/DІІ). (**C**) HPLC analysis of PT, PTF and LYC isomers. (**D**) The calculation method of %Ⅲ/Ⅱ.

**Figure A1:**
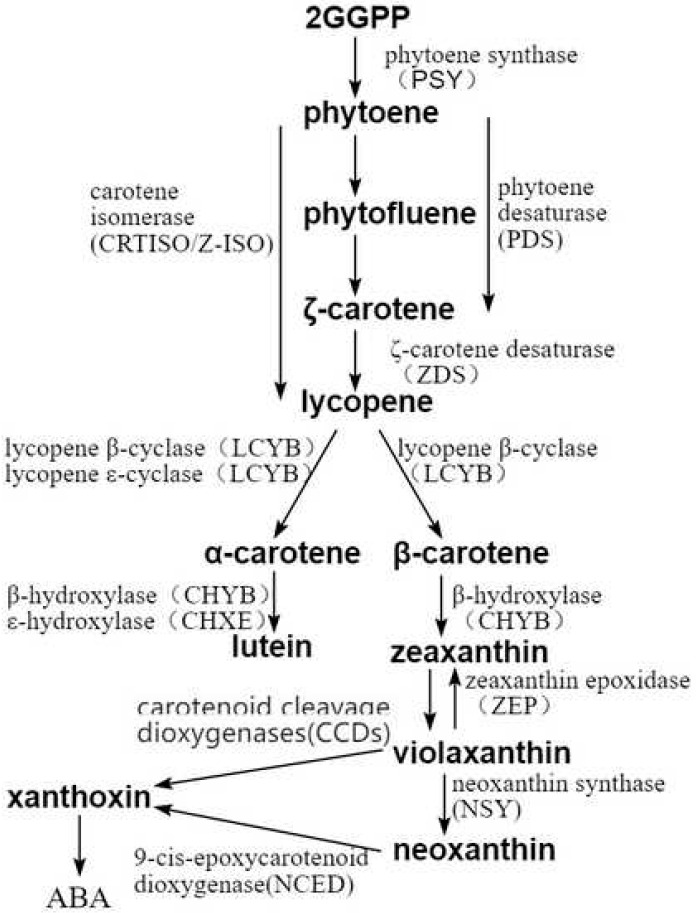
The biosynthetic pathway of carotenoids in plants.

Carotenoids are major food bioactive compounds in our diets, with antioxidant, anti-inflammatory and gut health functions [1]. The roles of carotenoids such as lycopene (LYC), astaxanthin and β-carotene in preventing chronic diseases and inhibiting cancer have been widely accepted [2,3,4]. Meanwhile, phytoene (PT) and phytofluene (PTF), known as the precursors in carotenoid synthesis (Figure A1), and commonly called polyhydrogen lycopene, have attracted more and more attention [5,6,7,8]. PT and PTF are C40 isoprenoid compounds with fewer conjugated double bonds than downstream metabolites [9] (PT, PTF, LYC and β-carotene contain 5, 3, 11 and 11 conjugated double bonds, respectively); their molecular structures are shown in Figure 1, and the physicochemical properties of PT and PTF are different from those of other carotenoids [10]. For example, both PT and PTF absorb light in the ultraviolet region of the spectrum, with maximum absorption wavelengths at 286 nm and 348 nm, respectively. Therefore, they are colorless in the visible region [11]. The daily intake of PT and PTF constitutes approximately one fifth of the dietary carotenoids [12,13] and can increase the content in the body through exogenous intake [6]. PT and PTF have high bioavailability [14,15] and may be the contributing components of UV protection in the skin [11,16]. Many studies have proven that PT, PTF and LYC may have synergistic antioxidant effects and potential mechanisms for alleviating and preventing corresponding diseases [6,7,8].

Tomato fruit and its products are a major dietary source of LYC and other carotenoids, which are sensitive to heat, light, oxygen and pH, and might be lost due to isomerization and oxidative degradation during processing [17]. Tomato powder has a wide range of applications. It is conducive to storage and transportation, and especially has a higher concentration of effective substances. For example, tomato powder is usually used as a material with remarkable effects in some scientific studies [18]. β-carotene and LYC are the carotenoids that are often of concern in studies related to tomato powder drying [19,20]. Several studies have found an increase in β-carotene and LYC content in tomato products after drying treatment compared to fresh tomatoes, and have recognized that this proportion of increase is due to changes in extractability [20]. However, some studies have found that different conditions and parameters used in drying treatments may lead to different levels of LYC degradation [21]. Due to the incomplete understanding of the drying stability of PT, PTF and LYC, it seems that FD technology is the most reliable method, which can maximize the retention of bioactive compounds, but it is time-consuming and energy-consuming [22], and it is not conducive to the retention of LYC [19]. Staged drying seems a good way to preserve carotenoids, such as adjusting parameters in a single drying process or using two different drying technologies in succession. A study shows that in HAD technology, the combination of 90 °C (2 h) + 50 °C (48 h) yields the highest increase of LYC, up to 2.3 times [19]. Siebert [22] investigated the development of quality parameters (volume retention, rehydration properties) during FD, HAD and microwave vacuum drying, either as a single or as a serial combination process with a varying changeover point. The combined drying process (CDP) can achieve the desired product quality parameters, partially with shorter drying times. It seems that these methods can provide a reference for the selection of drying methods of tomatoes. Therefore, hot-air vacuum drying technology (CDP) was selected in this study, and the effects of different process parameters on the retention rates of PT, PTF and LYC were studied.

Although there are several studies on the effect of carotenoid content during the drying process, such as LYC, there is still a lack of systematic studies [19]. In particular, most studies on the drying process of tomato powder have not considered the loss of PT and PTF content [23]. The stability between polyhydrogen lycopene and LYC during processing might be different. On the one hand, during processing, LYC forms crystalline structures in the tomato with higher concentrations of LYC, and it is more stable. However, there is no information on the possible deposition forms of PTF [24]. On the other hand, in tomatoes, the dominant isomers of PT, PTF and LYC are different ((15-cis)-PT, (All-*E*)-PTF and (All-*E*)-LYC, respectively), and the stability of (15-cis)-PT, (All-*E*)-PTF and (All-*E*)-LYC is also different, which may also contribute to differences in their processing stability [25]. The authors believe that most of the studies concerned with the processing stability of PT and PTF were conducted in a tomato matrix with high water content, such as tomato juice and tomato pulp. These studies generally agree that PT and PTF in the tomato matrix have good processing stability [14,26,27]. However, this conclusion is not consistent with the structural characteristics of PT and PTF in theory [21]. Although there are already a few PT- and PTF-enriched products on the market, such as whitening nutricosmetics and capsules [5], the innovative product development potential is mostly untapped [28]. Therefore, it is necessary to study the retention rate changes of PT and PTF, including LYC, in drying processing.

The main objective of this research was to design a feasible drying method to increase the retention rates of PT, PTF and LYC in tomato powder. Firstly, the tomato raw material rich in PT, PTF and LYC was screened. Furthermore, we explored and compared the effects on the retention rates in different drying methods and conditions under vacuum drying. Finally, a feasible drying method (CDP) was designed and parameters were selected from the perspective of improving the retention rates of PT, PTF and LYC. Some other parameters related to the quality of tomato powder are also provided, such as 5-hydroxymethyl-2-furaldehyde (5-HMF) and so on.

## 2. Materials and Methods

### 2.1. Main Materials and Instruments

Seventeen varieties of tomato, mainly from the China (Shouguang) International Vegetable Science and Technology Expo or the vegetable base in Weifang (Shandong, China). Standards: Phytoene (97.0% CaroteNature), phytofluene (99.2% CaroteNature), (All-*E*)-Lycopene (HPLC ≥ 95% Pureone), 5-HMF (>99% Shanghai Aladdin Bio-Chem Technology Co., Ltd., Shanghai, China). Reagents: Hexane, acetone, methanol, ethyl acetate, potassium chloride (KCl), zinc sulfate heptahydrate (ZnSO_4_⋅7H_2_O), copper (II) sulfate pentahydrate (CuSO_4_⋅5H_2_O), calcium chloride anhydrous (CaCl_2_), magnesium chloride anhydrous (MgCl_2_), aluminum chloride (AlCl_3_), iron (III) sulfate hydrate (Fe_2_(SO_4_)_3_), iron (II) sulfate heptahydrate (FeSO_4_). All the above were analytically pure, purchased from Sinopharm Chemical Reagent Co., LTD. (Shanghai, China). HPLC-grade solvents for chromatography, including acetonitrile (ACN), methanol and methyltert-butyl ether (MTBE), were purchased from Oceanpak (Shanghai, China), and ultrapure water was used for the experiment.

### 2.2. Material Pretreatment

Fresh tomatoes were cleaned, cut into pieces, crushed in a blender for 30 s, sieved through a 1.18 mm sieve only to remove the peel and seeds, and finally formed a pulp. After detecting the content of PT, PTF and LYC in different varieties of tomatoes, the varieties (red tomato NO.9 and yellow tomatoes NO. 1, 2, 4, 5) with higher content of PT and PTF were selected and mixed together to form tomato pulp. This helped to obtain sufficient raw material with a detectable level of polyhydrogen lycopene (PT and PTF) for the subsequent experiments, and the content is shown in Table A1.

### 2.3. Different Drying Methods

First, 30 g tomato pulp was evenly dispersed in several glass Petri dishes with a diameter of 9 cm and dried under the following drying conditions. The process parameters were determined based on pre-experiments. (1) Vacuum freeze-drying (FD) (ALPHA 1–2 LD plus, Christ, Germany): The tomato pulp was pre-frozen overnight at −80 °C and then transferred to a preheated vacuum freeze dryer. The parameters were set to −54 °C, 0.52 mbar, and the total drying time was approximately 30 h. (2) Vacuum drying (VD) (TCHZ-6020, Tongke, Shanghai, China): The temperature was 80 °C. The degree of vacuum was 0.1 mPa. Drying time was approximately 4 h. (3) Hot-air drying (HAD) (Yuejin, Shanghai, China): The temperature was 80 °C. The drying time was approximately 5 h. Tomato variety screening was carried out under the above vacuum freeze-drying conditions. The drying terminal point was 5 ± 2% water content.

### 2.4. The Effect of Temperature, pH Value and Metal Ions under Vacuum Drying

The drying effect under different VD conditions on the retention rates of PT, PTF and LYC was investigated. Before drying, the tomato pulp was preheated in a 60 °C water bath for 10 min, and then quickly weighed and distributed to the corresponding Petri dishes. Under the condition of a vacuum degree of 0.1 mpa, the effect of different VD temperatures (60 °C, 65 °C, 70 °C, 75 °C, 80 °C, 85 °C) on the retention rates was investigated. The effect of the initial pH (the pH value of tomato juice will change slightly during the drying process; the initial pH refers to the pH value of fresh tomato raw material before drying) of tomato pulp on the retention rates was studied under VD conditions of 80 °C and 0.1 mPa. The pH value (pH 3, pH 4.5, pH 6, pH 7.5, pH 9) was adjusted by 2.5 mol/L HCl and 1 mol/L NaOH. Under VD conditions of 80 °C and 0.1 mPa, the effect of several metal ions (Fe^3+^, Zn^2+^, Mg^2+^, Ca^2+^, Fe^2+^, Cu^2+^, K^+^) commonly added in food processing on the retention rate was studied. The metal ions mentioned above were added to tomato pulp at the concentration of 1 mmoL/L, respectively (refer to Wang [29] for the added amount).

### 2.5. Combined Drying Process

The tomato powder was prepared by hot-air vacuum drying (CDP) technology, i.e., when the water content of the tomato pulp was relatively high, the tomato pulp was dried by HAD to the changeover water content [30], and then was transferred to VD. Before drying, the tomato pulp was preheated in a 60 °C water bath for 10 min, and then quickly weighed and distributed to the corresponding Petri dishes. The changeover water content, HAD temperature, VD temperature, VD degree and loading weight (different liquid layer thickness) were determined successively. During the drying process, the water content was controlled within the allowable 2% error, and the obtained tomato powder was pulverized through a 0.85 mm sieve. All samples were sealed in aluminum foil bags and stored in a −20 °C refrigerator until extraction.

### 2.6. Water Content and Retention Rate

The initial water content was determined by the “National Food Safety Standard—Determination of Water Content in Foods (China)” [31].

The change in water content during drying was determined as follows:

(1) $$W=\frac{W_{1}-W_{2}}{W_{1}}\times 100$$

where W is the water content of the sample, %; W_1_ is the initial mass of the sample, g; W_2_ is the mass of the sample during drying or the mass of dry powder, g.

(2) $$\mathrm{Carotenoid}\;\mathrm{retention}\;\mathrm{rate}\left(\%\right)=\frac{{\mathrm{DW}}_{\mathrm{after}}}{{\mathrm{DW}}_{\mathrm{before}}}\times 100$$

where DW_after_ is the carotenoid content of tomato powder or puree after drying treatment, ug/g dry weight; DW_before_ is the carotenoid content in tomato pulp, ug/g dry weight.

### 2.7. Carotenoid Extraction

Carotenoid extraction from the tomato [24]: Approximately 0.5 g dry matter content of the tomato powder or other tomato samples was added into a triangle beaker with 20 mL extraction solvent: n-hexane: methanol: acetone (50:25:25, mL/mL/mL). It was then stirred with a magnetic stirrer for 20 min. The supernatant was collected by vacuum filtration, and the residue was filtered and extracted twice until discoloration. The supernatant was combined, and the carotenoid-containing organic phase was separated with a separating funnel and then concentrated with a rotary evaporator at 40 °C and 250 mbar. The concentrate was dissolved in ethyl acetate and diluted to the appropriate concentration with methanol: MTBE (50:50, mL/mL). The solution was filtered through a 0.22 μm polytetrafluoroethylene membrane filter (PTFE).

### 2.8. Quantification and Isomer Determination

Isomers of PT, PTF and LYC were analyzed by reversed-phase HPLC with a diode array detector (Alliance 2695, Waters Corp, Shanghai, China). Separation was performed on a polymeric C30 column (YMC carotenoids, 5 μm, 250 × 4.6 mm, Shanghai, China). The column temperature was 30 °C, and the injection volume was 0.02 mL. The HPLC method was developed by Cooperstone et al. [24]. The quantification of PT, PTF and LYC isomers was performed by peak area determination at 286, 348 and 471 nm, respectively, and by an external standard curve. The identification was conducted by retention time comparison to standards and spectroscopic features (Figure 1, Table 1).

### 2.9. pH Value, Color and Power Consumption

pH values of tomato pulp or tomato pulp with adjusted pH values were measured by a pH meter (Mettler Toledo, Zurich, Switzerland). Ten tomatoes of each variety were selected randomly, and then three points of each tomato were selected along the equatorial plane. Tomato powder obtained by CDP was packed in a transparent sealed container, and three points were selected uniformly. It was measured by a colorimeter (NR60CP, Shien, Shenzhen, China), and the color parameters were expressed in the color space CIELAB (L*, a*, b*, H°). The power consumption of tomato powder obtained by CDP technology was calculated based on the recorded drying time (Table A4) and the usage of the instrument nameplate.

### 2.10. 5-HMF and A420

5-HMF extraction [33]: A volume of 0.5 g tomato powder obtained by CDP was added to 10 mL of 50% methanol solution. After 3 h of magnetic stirring at room temperature, it was filtered through a 0.22 µm PTFE membrane filter. The filtrate was diluted 5 times with 50% methanol–water before detection. The extract could also be used to measure absorbance at 420 nm, as an indicator of Amadori compound browning (A_420_) [34]. Next, 5-HMF was quantified using an ultra-high-performance liquid chromatography–tandem mass spectrometry (UPLC–TQD) system + ESI mode. The injection volume was 1 μL. Separation was performed on an ACQUITY C18 (1.7 μm, 2.1 × 100 mm) column, 0.2 mL/min. The mobile phase elution procedure referred to Yang [33]. MS detection was accomplished using multiple reaction monitoring (MRM) analysis (Table A2). The MS/MS analysis was in the range of 100−200 m/z. Quantitation of 5-HMF in the samples was accomplished by the 5-HMF standard curve.

### 2.11. Statistical Analysis

Each sample was extracted in triplicate. The experiments were repeated at least two times for verification. Duncan’s multiple comparisons were used for comparisons of the means, and statistical significance tests were conducted at the level of 0.05 (*p* < 0.05). One-way ANOVA was used for significance analysis, and the significant differences were calculated at the level of 0.05 (*p* < 0.05) (SPSS 26.0, IBM, Chicago, IL, USA). Results are expressed as mean ± standard deviation (SD). Origin 2018 and Indraw were used for graphs and charts.

## 3. Results and Discussion

### 3.1. Selection of Tomato Varieties

**Figure 2:**
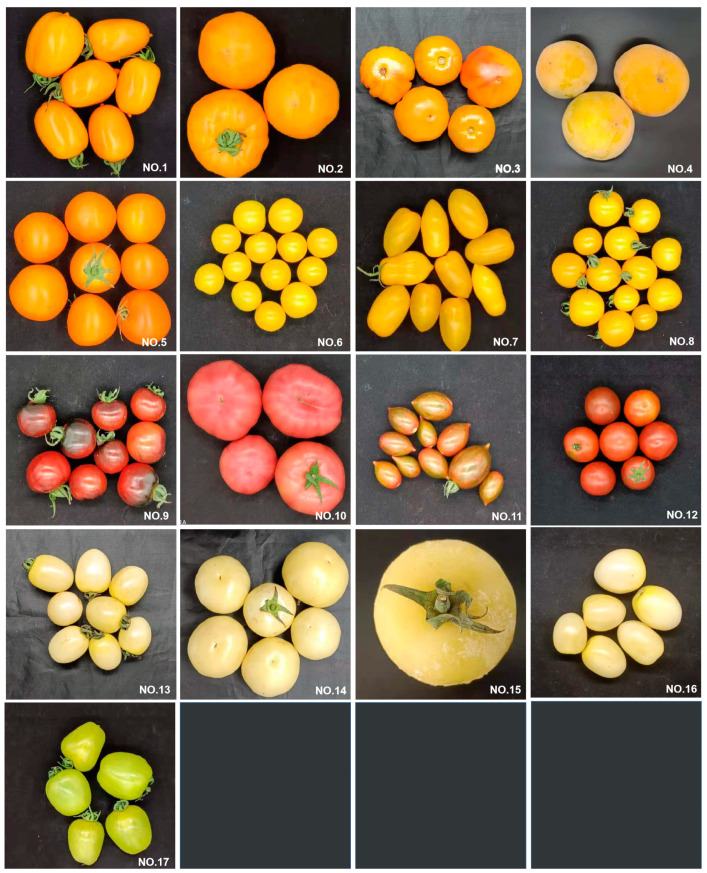
The appearance of tomato varieties.

The PT, PTF and LYC content of 17 tomato varieties (number form) was detected (Table 2). The appearance diagrams are shown in Figure 2. The five varieties with the highest polyhydrogen lycopene levels (total content of PT and PTF) were No. 5, 2, 9, 1 and 4, from high to low. Among them, the dry weight content of polyhydrogen lycopene in No. 5 was the highest, reaching 0.06%. The content of PTF in No. 2 was four-times higher than that of PT at the mature stage. This ratio was reported for the first time. Our results can provide a basis for further subdividing sources of agricultural substances of PT and PTF in the future. In order to reveal the possible correlations between the appearance color of tomato varieties and the content of PT, PTF and LYC, the color parameters (L*, a*, b*, H°) of No. 5, 2, 9, 1 and 4 are shown in Table A3. The a* parameter (axis of red–green) ranged from 1.91 up to 5.91. The b* parameter (axis of yellow–blue) ranged from 6.92 in red tomato (No. 9) up to 12.75 in orange tomato. The L* (lightness) parameter ranged from 44.25 to 51.43. The H° angle parameter varied from 56.06 in red tomato (NO.9) up to 81.69 in orange tomato. With the exception No. 9, which was a special red variety, all varieties were orange. The content of PT and PTF and color characteristics in tomatoes were similar to those reported in the literature [14,35]. Some studies have shown that differences in apparent fruit color are correlated with carotenoid accumulation [36]. The characteristics of orange tomatoes were similar to those of citrus tomatoes produced by carotenoid isomerase (CRTISO) mutation [37]. White, green and light yellow tomatoes may not accumulate enough pyrophosphate (GGPP) or PT due to abnormal expression of the PSY1 gene [38]. With the exception of the variety, factors such as climate, soil moisture and nutrients, harvest time and maturity status also affect the accumulation of polyhydrogen lycopene [12].

### 3.2. Different Drying Methods

**Figure 3:**
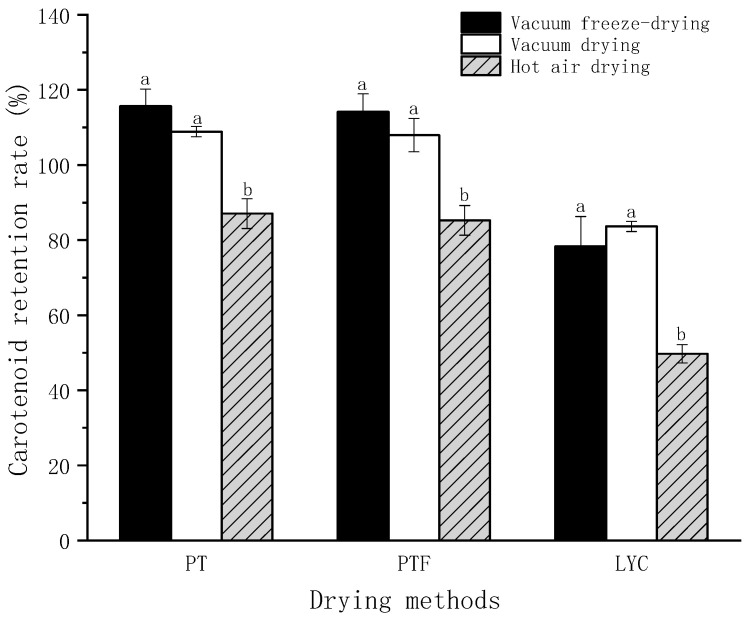
The retention rates of PT, PTF and LYC in different drying methods. The raw material was the mixed tomato pulp of five tomato varieties selected. The water content terminal point 5 ± 2%. The retention rate was obtained by comparing tomato powder with tomato pulp material. Different letters of the same carotene indicate significant differences, according to Duncan test (*p* < 0.05).

The retention rates of FD, VD and HAD technology to the terminal point (5 ± 2%) were compared under the aforementioned parameters, and the results are shown in Figure 3. The raw material was the mixed tomato pulp of the five tomato varieties selected. Among the three drying methods, FD had the best effect on the retention rates of PT, PTF and LYC. Compared with this, VD had no significant effect on the retention rates of PT, PTF and LYC. Preliminary results showed that under vacuum conditions, PT, PTF and even LYC in the tomato matrix had good thermal stability and could accept a certain degree of thermal processing. However, the retention rates of PT, PTF and LYC were reduced by around 29% in HAD compared with FD (PT from 20.05% to 37.15%, PTF from 20.10 to 37.62, LYC from 18.25% to 38.99%). Although some studies have found that the content of LYC in the product after HAD is higher than that of the original fresh tomato pulp [21,39], it is believed that HAD has little effect on the lycopene content of tomatoes. In fact, the unextracted PT, PTF and LYC (approximately 30%) in fresh tomato pulp has actually been consumed, which can be seen by comparing the different drying methods. The set temperature of both HAD and VD was 80 °C, but the retention rates under VD were significantly higher than those under HAD. In the process of HAD, oxygen might be the most important factor in the decline in retention rate.

**Figure 4:**
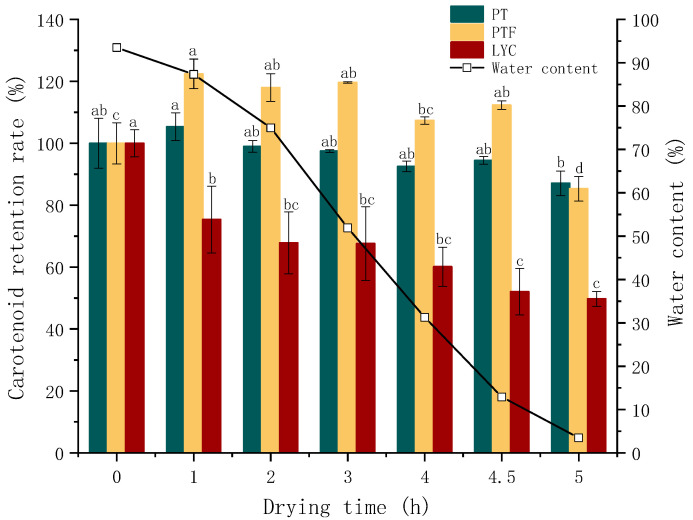
The change in retention rate and moisture content in hot-air drying (HAD). The raw material was the mixed tomato pulp of five tomato varieties selected. The retention rate was obtained by comparing tomato powder or puree after drying treatment with tomato pulp material. Different letters between columns of the same color are significant (*p* < 0.05), according to Duncan test.

Therefore, the changes in the PT, PTF and LYC retention rates during HAD were monitored (Figure 4). The stability of PT, PTF and LYC during HAD was related to the change in water content. When the water content decreased from 30% to 3.5%, the retention rates of PT, PTF and LYC decreased significantly (PT from 97% to 87%, PTF from 120% to 85%, LYC from 68% to 50%), and the decrease was much higher than that in the first 4 h (almost no decrease was observed). Zhang [21] found that under HAD, after the water content reached 15%, the LYC content decreased significantly. It was speculated that at low water content, PT, PTF and LYC lost their water packets and were more easily oxidized when exposed to air [40]. Lavelli [41] found that LYC was less stable in by-products of low water or water activity, suggesting that, in parallel, both cis and trans isomers of LYC auto-oxidize, forming volatile fragments. The effects of thermal treatment at 80 °C on the total LYC concentration in the water-based and oil-based samples were very similar, provided that the oil had a good ability to isolate oxygen. Therefore, when the heating temperature was lower or slightly higher than 80 °C, water could compete with oxygen for the active absorption sites [42]. The decrease in LYC retention was always accompanied by the drying process, and the decrease was more significant than that of PT and PTF. LYC was affected by both oxygen and temperature [24] and was more prone to isomerization, which was usually the first step in degradation [43]. In addition to water content, other antioxidant components in tomato may also play a role in PT, PTF and LYC resistance to oxidation [21].

Sometimes, the retention rates in the data were larger than 100%, which did not mean that new carotenoids were generated during the drying process. Because carotenoid synthesis requires the participation of enzymes in tomatoes [9], the drying process has broken this condition. The retention rate was obtained by comparing the carotenoid content of processed tomato powder with that of fresh tomato pulp. However, the extraction effect of PT, PTF and LYC from fresh tomato pulp was inferior to that of tomato powder. Extensive literature suggests that cell walls and chromoplasts (i.e., the carotenoid-containing plastids) appear as the major barriers limiting carotenoid diffusion [44]. The process has a positive impact on carotenoid release from fruit and vegetables, with the loss of the food matrix structure [18,20,41]. The authors believe that the plant cells in the fresh tomato pulp were relatively intact and fully packed with water, which affected the penetration of organic reagents into the plant cell structure to dissolve carotenoids. Therefore, the extracted carotenoid content of fresh tomato pulp was lower than that of dry powder. Fortunately, most of the samples in this research were extracted uniformly in dry powder form, which did not affect the conclusion [42].

### 3.3. Vacuum Drying Temperature, Initial pH Value of Tomato Pulp and Metal Ions

**Figure 5:**
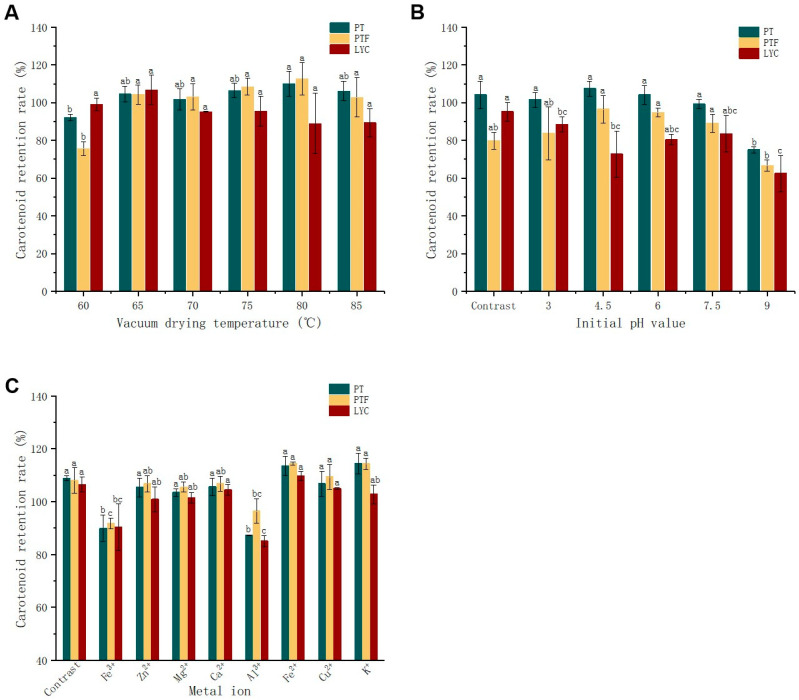
The effects of different conditions on retention rates of PT, PTF and LYC under vacuum drying. (**A**) The effects of different vacuum drying (VD) temperatures under 0.1 mpa; (**B**) different initial pH values at 80 °C and (**C**) 0.1 mPa and different metal ions with a concentration of 1 mmol/L at 80 °C and 0.1 mPa on the retention rates of tomato powders. Tomato powder dried directly from untreated tomato pulp was used as a control. The raw material was the mixed tomato pulp of five tomato varieties selected. The retention rate was obtained by comparing tomato powder with tomato pulp material (preheated in a 60 °C water bath for 10 min). Different letters between columns of the same color are significant (*p* < 0.05), according to Duncan test.

The effects of different VD temperatures at 0.1 mpa, different initial pH values of tomato pulp at 80 °C and 0.1 mPa and different metal ions with a concentration of 1 mmol/L at 80 °C and 0.1 mPa on the retention rates of tomato powders are shown in Figure 5. The raw material was the mixed tomato pulp of the five tomato varieties selected. There was no significant difference in the retention rates of PT, PTF and LYC at the selected temperatures, 65 °C (920 min), 70 °C (453 min), 75 °C (267 min), 80 °C (240 min), 85 °C (187 min), showing the good thermal stability of PT, PTF and LYC [14,24]. When the drying temperature reached 85 °C, the retention rates of PT, PTF and LYC in tomato powder were 106%, 103% and 89%, respectively. Ma [27] and Yu [45] added sulfur-containing compounds, which can lead to the isomerization of PT, PTF and LYC. At the same time, heat treatment was carried out, and no change in content was found. A study compared the thermal stability of PT, PTF, LYC and other carotenoids in orange juice and a simulated system heated at 100 °C for 600 min. The content of PT, PTF and LYC in the simulated system was reduced by approximately 12.50%, 25% and 70.85%, respectively, but there was no change in the fresh orange juice [46]. The above results indicated that the tomato matrix structure, blocking carotenoid extraction, might have a protective effect on PT, PTF and LYC [44].

During the VD process, when the pH value was 9, the retention rates of PT, PTF and LYC in tomato powder (5 ± 2%) decreased by 29%, 13% and 33%, respectively, compared with the raw tomato pulp (pH 4.28) dried to tomato powder directly without pH treatment. In a strongly alkaline environment, LYC experienced anionic polymerization and precipitation. The structures of PT, PTF and LYC were close, suggesting that they might have similar reactions [47]. Fortunately, PT, PTF and LYC are rarely used in pH > 7 food systems.

Moreover, 1 mmol/L Fe^3+^ and Al^3+^ significantly reduced the retention rates of PT, PTF and LYC during VD of tomato pulp. Fe^3+^ reduced the retention rates of PT, PTF and LYC by 19%, 16% and 16%, respectively, compared with the raw tomato pulp dried to tomato powder directly, without the addition of metal ions. Al^3+^ reduced the retention rates of PT, PTF and LYC by 22%, 12% and 21%, respectively. However, its effect was lower than that in acetone solution. Cu^2+^ and Fe^2+^, which were reported to work in acetone solution, did not affect the drying process of tomato powder. Although the calculated concentrations of LYC were similar compared to those reported [29], AlCl_3_ catalyzed LYC as a whole molecule rather than in ion form. AlCl_3_ is a typical Lewis acid that can activate olefins or alkynes by forming π complexes [29]. Therefore, it was speculated that the tomato matrix can also protect against the degradation of PT, PTF and LYC by metal ions. Fe^3+^ and Al^3+^ should be avoided in tomato powder processing [24].

### 3.4. Combined Drying Process

**Figure 6:**
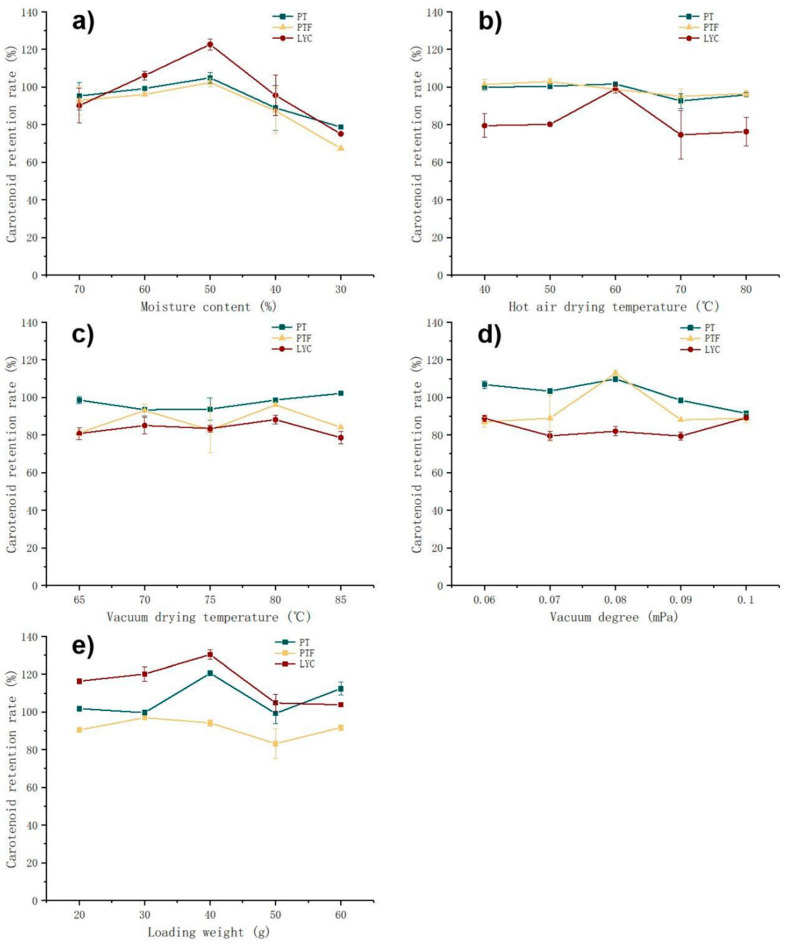
The influence of changeover water content, HAD temperature, VD temperature, VD vacuum and loading weight on retention rates is shown in (**a**–**e**), respectively. Besides the parameters explored, other parameters were as follows: (**a**) HAD temperature 60 °C, VD temperature 80 °C, VD vacuum 0.1 mPa and loading weight 30 g; (**b**) MTP 50%, VD temperature 80 °C, VD vacuum 0.1 mPa and loading weight 30 g; (**c**) changeover water content 50%, HAD temperature 60 °C, VD vacuum 0.1 mPa and loading weight 30 g; (**d**) changeover water content 50%, HAD temperature 60 °C, VD temperature 80 °C and loading weight 30 g; (**e**) changeover water content 50%, HAD temperature 60 °C, VD temperature 80 °C and VD vacuum 0.08 mPa. The raw material was the mixed tomato pulp of five tomato varieties selected. The retention rate was obtained by comparing tomato powder with tomato pulp material (preheated in a 60 °C water bath for 10 min).

According to the earlier research results (Figure 3), compared with FD technology, VD technology did not significantly reduce the retention rates of PT and PTF in tomato powder. Compared with the higher cost of FD, it was easier to realize industrial production. At the same time, it was found that in HAD, the retention rates of PT and PTF changed significantly only when the water content decreased to a certain value (Figure 4). According to existing literature, through the serial combination of hot air and freeze drying, LYC and β-carotene were well preserved and the product quality was improved [48]. Therefore, a serial combination of hot-air and vacuum drying (CDP) was designed to produce tomato powder. The raw material was the mixed tomato pulp of the five tomato varieties selected. The influences of changeover water content, HAD temperature, VD temperature, VD degree and loading weight on the retention rates are shown in Figure 6.

Meanwhile, the production parameters of tomato powder were also determined, referring to 5-HMF, A_420_, power consumption, time and color parameters (Table A4). Acrylamide was not detected in any samples and no further analysis was performed [49]. According to the previous conclusions, changeover water content was the most important parameter to be determined first. When the changeover water content was below 50%, the retention rates of PT, PTF and LYC were significantly reduced. When the changeover water content was 30%, the retention rate of PT and PTF decreased by nearly 30%, and the retention rate of LYC decreased by nearly 50% compared with a changeover water content of 50%. Under the protection of the tomato matrix with high water content (changeover water content 70% and 60%), the retention rates of PT and PTF did not change significantly, because oxygen was isolated by water and the effect of heat on PT was relatively weak.

After selecting a suitable changeover water content, the HAD temperature and VD temperature did not significantly change the retention rates. Combined with 5-HMF, A420 and other parameters (Table A4), HAD temperature 60 °C [50] and VD temperature 80 °C [51] were determined as the most suitable process conditions, which were close to the reported suitable parameters. The data suggested that a VD degree below 0.08 mPa was conducive to PT retention. Heat might cause the tomato tissue to shrink [20]. When the loading weight of each batch was 20 g, 30 g, 40 g, 50 g and 60 g, the thicknesses of the sample in the Petri dish with a diameter of around 94 mm were 2.88 mm, 4.06 mm, 5.70 mm, 7.88 mm and 8.08 mm, respectively. The shrinkage of the upper tissue has a certain protective effect on PT, PTF and LYC in the lower tissue. However, an excessive increase in loading weight would prolong the heating time and reduce the retention rates of PT, PTF and LYC. Compared with PT and PTF, the retention rate of LYC was more unstable under the influence of multiple parameters; this was because, in terms of stability, PT > PTF > LYC [15]. The results showed the final parameters: changeover water content was 50%, HAD temperature was 60 °C, VD temperature was 80 °C, VD degree was 0.08 mPa and the loading weight was 40 g (thickness 5.70 mm). The retention rates of PT, PTF and LYC were 121%, 94% and 130%, respectively. Compared with VD alone, the CDP technology improved the retention rates of PT and LYC by 12% and 36%, respectively, while PTF decreased by only 6% (Figure 3). This was because part of the VD at 80 °C was replaced by HAD at 60 °C.

Moreover, 5-HFM and A_420_ were the symbolic substances used to judge the Maillard reaction process, and they are also harmful substances that might be produced in the drying process. From the data in Table A4, both were at low levels. From the max–min, the water content and vacuum degree were the most obvious factors affecting the formation of 5-HMF, while the vacuum drying temperature and vacuum degree were the most obvious factors affecting the A_420_ value [52]. A high value of a* indicated that the color of the tomato powder was reddish, and high values of L* and b* indicated that the color of the tomato powder was yellowish (Table A4) [53]. Generally speaking, tomato powder with a significantly higher A_420_ had a lower a* value.

## 4. Conclusions

In this study, tomato raw material rich in PT, PTF and LY was obtained. When comparing the three drying methods, only the HAD technology caused an obvious decrease when the water content was below 30% (PT from 97% to 87%, PTF from 120% to 85%, LYC from 68% to 50%). We speculated that the stability of PT, PTF and LYC during HAD was related to the water content. Then, the drying properties of PT, PTF and LYC under VD were discussed. It was found that alkaline conditions (pH = 9), Fe^3+^ and Al^3+^ could significantly reduce the retention rate (*p* < 0.05). The total retention rates of polyhydrogen lycopene decreased by 22%, 12% and 7% under these conditions, respectively. The retention rates of LYC decreased by 33%, 15% and 17% under these conditions, respectively. The temperature did not change the retention rates significantly (*p* < 0.05; at 85 °C, the retention rate of polyhydrogen lycopene was still 104%). Finally, in order to ensure the retention rates of PT, PTF and LYC, the CDP was designed and the suitable parameters were determined, where the retention rates of PT, PTF and LYC were 121%, 94% and 130%, respectively. This article mainly provides a feasible drying technology for producing tomato powder rich in PT, PTF and LYC and with good inspection indexes.

## Figures and Tables

**Table 1:** Identification of PT, PTF and LYC isomers.

Carotenoids	Retention Time (Min) Observed	λmax (In-Line) (nm)	%Ⅲ/Ⅱ ^a^		Q Value ^b^	
			In-Line	Reported	In-Line	Reported
(Z1)-PT	6.34	286	11.14	-	-	-
(15Z)-PT	9.29	285.5	16.38	-	-	-
(All-*E)*-PT	ND	ND	ND	19.05 ^c^, 9.09 ^d^	-	-
(Z1)-PTF	10.35	332, 347, 364	61.09	52.20 ^c^, 70.50 ^d^	-	-
(Z2)-PTF	ND	ND	ND	82.20 ^c^, 78.60 ^d^	-	-
(Z3)-PTF	ND	ND	ND	89.30 ^c^, 80.60 ^d^	-	-
(All-*E)*-PTF	11.93	331, 347, 364	84.86	91.40 ^c^, 82.61 ^d^	-	-
(Z4)-PTF	ND	ND	ND	90.30 ^c^, 78.90 ^d^	-	-
(13Z)-LYC	29.33	360, 439, 464, 495	-	-	0.53	0.58 ^d^, 0.58 ^e^
(9Z)-LYC	31.27	360, 441, 467, 497	-	-	0.15	0.14 ^d^, 0.14 ^e^
(All-*E*)-LYC	33.50	361, 445, 472, 502	-	-	0.07	0.06 ^d^, 0.07 ^e^
(5Z)-LYC	33.90	361, 445, 472, 502	-	-	0.07	0.06 ^d^, 0.07 ^e^

Values and peak designations were obtained from the chromatograms of the samples. ^a^ Taking the minimum absorbance value between the two peaks as the baseline, the peak height ratio of the right peak and the left peak relative to the baseline is expressed in the form of a percentage. ^b^ Ratio of the maximum height of the *Z* peak (DB) to the maximum height of the main absorption peak (DII). ^c, d, e^ identified according to previous work in reference [27,29,32] respectively.

**Table 2:** The content of PT, PTF and LYC in 17 tomato varieties.

Tomato Variety Number	PT Content ug/g DW	PTF Content ug/g DW	LYC Content ug/g DW
No. 1	91.10 ± 6.42 ^d^	275.70 ± 15.02 ^b^	143.77 ± 0.45 ^c^
No. 2	83.53 ± 4.62 ^d^	413.04 ± 7.92 ^a^	20.65 ± 0.35 ^c^
No. 3	ND	ND	167.65 ± 1.98 ^c^
No. 4	167.11 ± 4.07 ^c^	93.19 ± 0.56 ^e^	104.17 ± 0.09 ^c^
No. 5	349.17 ± 39.25 ^a^	234.91 ± 6.98 ^c^	163.85 ± 2.33 ^c^
No. 6	ND	ND	67.98 ± 0.33 ^c^
No. 7	ND	ND	19.05 ± 0.09 ^c^
No. 8	ND	ND	20.57 ± 0.23 ^c^
No. 9	249.94 ± 16.75 ^b^	156.58 ± 11.36 ^d^	1145.81 ± 2.14 ^a^
No. 10	73.28 ± 8.71 ^de^	22.66 ± 1.40 ^g^	768.28 ± 1.10 ^b^
No. 11	36.58 ± 5.77 ^ef^	18.99 ± 2.32 ^gh^	1236.80 ± 3.99 ^a^
No. 12	110.58 ± 2.81 ^d^	44.83 ± 0.43 ^f^	1269.15 ± 250.49 ^a^
No. 13	ND	ND	1091.35 ± 1.59 ^a^
No. 14	ND	ND	10.64 ± 2.20 ^c^
No. 15	26.18 ± 1.34 ^g^	2.16 ± 0.67 ^h^	24.15 ± 3.54 ^c^
No. 16	ND	ND	ND
No. 17	ND	ND	ND

Data listed in the table were tested in triplicate and expressed as mean ± standard deviation. Different superscripts in the same column indicate significant differences, according to Duncan test (*p* < 0.05). ND indicates that the content is not detected.

## Data Availability

Data is contained within the article.

## Appendix A

**Table A1:** The content of PT, PTF and LYC in mixed raw material.

PT Content ug/g DW	PTF Content ug/g DW	LYC Content ug/g DW
170.19 ± 4.53	110.91 ± 0.97	1003.62 ± 15.01

Data listed in the table were tested in triplicate and expressed as mean ± standard deviation.

**Table A2:** UPLC–MS/MS performance of 5-HMF.

	Cone Voltage (eV)	Collision Energy (eV)	Molecular Ion	Quantitative *m*/*z*
5-HMF	30	15	127	127 → 81
	30	13		127 → 109

**Table A3:** The color parameters of No. 1, 2, 4, 5 and 9 tomato varieties.

Tomato Variety Number	Color	L*	a*	b*	H°
No. 1	orange	44.45 ± 3.29	3.78 ± 1.57	9.82 ± 1.88	69.50 ± 4.34
No. 2	orange	51.43 ± 2.96	4.25 ± 1.17	12.04 ± 3.12	70.59 ± 1.26
No. 4	orange	48.89 ± 1.50	1.91 ± 0.67	12.75 ± 2.45	81.69 ± 1.26
No. 5	orange	47.35 ± 2.02	5.91 ± 1.43	12.58 ± 2.61	64.83 ± 1.63
No. 9	red (with purple)	44.25 ± 2.64	4.73 ± 1.74	6.92 ± 2.26	56.06 ± 2.53

**Table A4:** The 5-HMF, A420, power consumption, drying time and color parameters of tomato powder.

Factor	Standard	5-HMF	A_420_	Power Consumption/kWh	Time/h	Color Parameters			
						L*	a*	b*	H°
Changeover water content	70%	15.31 ± 1.21 ^a^	0.10 ± 0.00 ^d^	11.96	6.83	45.42 ± 0.76 ^abc^	9.02 ± 0.54 ^a^	5.25 ± 0.39 ^a^	30.21 ± 1.37 ^a^
	60%	12.19 ± 1.14 ^b^	0.24 ± 0.01 ^c^	12.64	6.83	46.40 ± 0.36 ^c^	9.00 ± 0.07 ^a^	3.52 ± 0.16 ^b^	21.38 ± 0.88 ^b^
	50%	10.74 ± 0.85 ^b^	0.17 ± 0.01 ^e^	14.12	7.30	47.32 ± 0.64 ^a^	9.94 ± 0.65 ^a^	4.04 ± 0.11 ^b^	22.20 ± 0.85 ^b^
	40%	3.80 ± 0.09 ^c^	0.25 ± 0.00 ^b^	14.41	7.35	44.84 ± 0.40 ^ab^	5.55 ± 0.57 ^b^	3.58 ± 0.35 ^b^	32.84 ± 2.33 ^a^
	30%	5.24 ± 0.20 ^c^	0.38 ± 0.01 ^a^	16.79	8.08	46.09 ± 0.66 ^bc^	5.67 ± 0.14 ^b^	2.45 ± 0.19 ^c^	30.21 ± 1.37 ^a^
Hot-air drying temperature	40 °C	12.34 ± 1.56 ^a^	0.34 ± 0.01 ^a^	24.40	13.22	41.76 ± 0.05 ^a^	15.59 ± 0.46 ^a^	14.90 ± 0.44 ^ab^	30.21 ± 1.37 ^a^
	50 °C	13.37 ± 1.17 ^a^	0.33 ± 0.01 ^a^	17.73	10.55	41.48 ± 1.15 ^a^	13.78 ± 0.63 ^b^	12.57 ± 0.53 ^c^	21.38 ± 0.88 ^b^
	60 °C	10.74 ± 0.85 ^a^	0.17 ± 0.01 ^d^	13.56	8.88	41.32 ± 0.64 ^a^	13.94 ± 0.65 ^b^	14.04 ± 0.11 ^b^	22.20 ± 0.85 ^b^
	70 °C	6.63 ±0.11 ^b^	0.22 ± 0.02 ^c^	9.56	7.28	41.12 ± 0.43 ^a^	13.96 ± 0.37 ^b^	11.53 ± 0.62 ^c^	32.84 ± 2.33 ^a^
	80 °C	10.82 ± 0.70 ^a^	0.29 ± 0.01 ^b^	8.15	6.72	41.33 ± 0.49 ^a^	15.89 ± 0.72 ^a^	15.76 ± 0.67 ^a^	22.11 ± 0.29 ^b^
Vacuum drying temperature	65 °C	6.15 ± 0.10 ^b^	0.19 ± 0.00 ^c^	13.99	8.92	42.88 ±2.07 ^b^	8.54 ± 1.45 ^a^	3.71 ± 0.65 ^a^	43.71 ± 0.24 ^ab^
	70 °C	5.00 ± 0.42 ^c^	0.25 ± 0.01 ^b^	13.51	7.85	43.13 ± 0.56 ^b^	7.32 ± 0.80 ^ab^	3.38 ± 0.69 ^a^	42.38 ± 0.20 ^bc^
	75 °C	4.24 ± 0.25 ^a^	0.21 ± 0.01 ^c^	13.32	7.43	46.65 ± 1.39 ^a^	7.62 ± 0.42 ^a^	2.66 ± 0.38 ^a^	42.20 ± 0.85 ^c^
	80 °C	10.74 ± 0.85 ^a^	0.17 ± 0.01 ^d^	13.17	7.10	46.65 ± 0.56 ^a^	6.94 ± 0.65 ^ab^	3.38 ± 0.83 ^a^	39.53 ± 0.77 ^d^
	85 °C	8.58 ± 0.75 ^b^	0.60 ± 0.01 ^a^	13.07	6.87	46.11 ± 0.61 ^a^	5.66 ± 0.08 ^b^	2.30 ± 0.06 ^a^	44.76 ± 0.69 ^a^
Vacuum drying vacuum	0.06 mPa	10.44 ± 1.03 ^b^	0.57 ± 0.01 ^c^	14.31	7.98	44.76 ± 0.17 ^a^	6.99 ± 0.06 ^ab^	2.00 ± 0.28 ^b^	23.46 ± 0.72 ^a^
	0.07 mPa	16.10 ± 0.20 ^a^	0.79 ± 0.02 ^a^	14.41	8.20	45.01 ± 0.98 ^a^	6.59 ± 0.20 ^ab^	2.35 ± 0.07 ^b^	24.50 ± 2.20 ^a^
	0.08 mPa	5.09 ± 0.23 ^c^	0.63 ± 0.01 ^b^	14.28	7.90	44.97 ± 0.39 ^a^	6.44 ± 0.22 ^b^	11.09 ± 0.32 ^a^	19.15 ± 1.62 ^b^
	0.09 mPa	15.67 ± 1.84 ^a^	0.48 ± 0.01 ^d^	14.22	7.78	44.20 ± 0.82 ^a^	7.25 ± 0.64 ^a^	2.45 ± 0.37 ^b^	22.20 ± 0.85 ^ab^
	0.1 mPa	10.74 ± 0.85 ^b^	0.17 ± 0.01 ^e^	14.22	7.78	45.40 ± 0.36 ^a^	7.00 ± 0.07 ^ab^	2.52 ± 0.16 ^b^	22.09 ± 0.23 ^ab^
Loading weight	20 g	11.89 ± 0.94 ^a^	0.13 ± 0.00 ^c^	6.24	4.37	43.60 ± 0.58 ^ab^	10.74 ± 0.10 ^a^	4.96 ± 0.19 ^ab^	15.91 ± 1.97 ^b^
	30 g	10.64 ± 0.57 ^ab^	0.19 ± 0.01 ^b^	12.81	7.22	47.32 ± 0.64 ^a^	9.94 ± 0.65 ^ab^	4.04 ± 0.11 ^b^	19.64 ± 1.05 ^ab^
	40 g	8.99 ± 0.87 ^ab^	0.22 ± 0.00 ^c^	12.15	7.63	42.86 ± 0.88 ^c^	10.69 ± 0.11 ^a^	5.58 ± 0.13 ^a^	21.73 ± 0.59 ^a^
	50 g	10.93 ± 0.84 ^ab^	0.23 ± 0.00 ^d^	18.90	10.33	41.47 ± 2.34 ^c^	8.76 ± 0.74 ^b^	4.79 ± 0.52 ^ab^	18.78 ± 2.96 ^ab^
	60 g	8.33 ± 0.91 ^b^	0.21 ± 0.00 ^a^	37.07	19.43	45.82 ± 0.24 ^ab^	9.21 ± 0.80 ^b^	4.67 ± 0.88 ^ab^	21.38 ± 0.88 ^a^

Tomato powder produced using CDP technology was tested. The raw material was the mixed tomato pulp of five tomato varieties selected. Besides the explored parameters, other parameters are the same as in Figure 6. Different letters in the same column under the same factor indicate significant differences, according to Duncan test (*p* < 0.05).
